# Effectiveness and safety of secukinumab in ankylosing spondylitis: real-life data from Midlands Ankylosing Spondylitis Collaboration (MASC)

**DOI:** 10.1093/rap/rkad029

**Published:** 2023-03-08

**Authors:** Adeola Ajibade, Haridha Pandian, Nibha Jain, Latika Gupta, Ramasharan Laxminarayan, Arumugam Moorthy, Roshan Amarasena, Natasha Cox, Hem Sapkota, Girish Kakade, Srinivasa Elamanchi, Athiveeraramapandian Prabu, Elaf Al-Samaraaie, Nick Barkham

**Affiliations:** Rheumatology Department, Royal Wolverhampton Hospitals NHS Trust, Wolverhampton, UK; Rheumatology Department, Royal Wolverhampton Hospitals NHS Trust, Wolverhampton, UK; Rheumatology Department, University Hospitals of Leicester NHS Trust, Leicester, UK; Rheumatology Department, Royal Wolverhampton Hospitals NHS Trust, Wolverhampton, UK; Rheumatology Department, City Hospital, Sandwell and West Birmingham Hospitals NHS Trust, Birmingham, UK; Rheumatology Department, University Hospitals of Derby and Burton NHS Foundation Trust, Derby, UK; Rheumatology Department, City Hospital, Sandwell and West Birmingham Hospitals NHS Trust, Birmingham, UK; Rheumatology Department, The Robert Jones & Agnes Hunt Orthopaedic Hospital NHS Foundation Trust, Oswestry, UK; Primary Health Care Sciences, Keele University, Newcastle-under-Lyme, UK; Rheumatology Department, Royal Wolverhampton Hospitals NHS Trust, Wolverhampton, UK; Rheumatology Department, Royal Wolverhampton Hospitals NHS Trust, Wolverhampton, UK; Rheumatology Department, City Hospital, Sandwell and West Birmingham Hospitals NHS Trust, Birmingham, UK; Rheumatology Department, City Hospital, Sandwell and West Birmingham Hospitals NHS Trust, Birmingham, UK; Rheumatology Department, City Hospital, Sandwell and West Birmingham Hospitals NHS Trust, Birmingham, UK; Rheumatology Department, Royal Wolverhampton Hospitals NHS Trust, Wolverhampton, UK

Key message

- A real-world multicentre study demonstrates clear benefit in efficacy and safety of secukinumab in AS treatment.

Dear Editor, In recent years, more drug options have been approved for the treatment of AS, one of which is the IL-17A inhibitor secukinumab [1]. Secukinumab was approved for treatment of AS by NICE [2] in September 2016, following demonstration of its efficacy in a number of phase 3 clinical trials, including five multicentre trials [3–5]. Given that external generalizability of clinical trials is limited, real-world assessments are vital to audit the utility of such practice. We therefore conducted a multicentre real-world assessment of the efficacy and safety of secukinumab in patients with AS across centres in the UK.

This multicentre, retrospective record-based review involved the Midlands Ankylosing Spondylitis Collaboration, East & West Midlands, UK. It was a collaborative review involving five hospitals (The Royal Wolverhampton NHS Trust; Queen’s Hospital Burton; Leicester Royal Infirmary; Robert Jones and Agnes Hunt Hospital; and Sandwell and West Birmingham Hospitals NHS Trust), assessing patients seen from 2017 to 2019. This study was part of the mandatory audit requirement to be compliant with National Institute for Health and Care Excellence (NICE) Technology Appraisal Guideline [TA407].

The primary outcome of our study was treatment response, quantified using the BASDAI [6], spinal visual analogue scale (VAS) [7] and CRP at baseline and 16 weeks after commencement of secukinumab. Secondary objectives were to determine covariate effects of previous exposure to tumour necrosis factor inhibitor (TNFi) therapies, in comparison to patients who were TNFi naive.

Eligible subjects were adults ≥18 years of age, with diagnosed AS according to the Assessment of Spondyloarthritis International Society (ASAS) classification criteria [8], commenced on secukinumab for active disease, according to the National Institute for Health and Care Excellence (NICE) recommendations [2]. Participants included patients with previous exposure and inadequate disease response to TNFi therapies and those who were TNFi naive.

Data on patient demographics, co-morbidities, previous TNFi exposure, baseline and week-16 BASDAI, spinal VAS score, CRP and adverse events were extracted retrospectively from electronic patient records. These were collated in a centralized data repository, accessible to contributors within the six centres. Details on methodology have been included in Supplementary Data S1, available at *Rheumatology Advances in Practice* online. Adequate treatment response was defined as reduction in BASDAI by 50% of pretreatment score or ≥2 units and decline in spinal VAS by ≥2 cm, in accordance with NICE guidelines.

Of 105 patients (male-to-female ratio ∼2.5:1) with a mean age of 45.5 (14.3) years, nearly one-third (35, 33.3%) were biologics naive, and nearly half (Table 1) had at least one previous TNFi. At baseline, mean BASDAI was 7.05 (1.69), mean spinal VAS was 7.63 (1.59) and median CRP was 5.4 mg/dl (3.0, 18.0). Based on the NICE response criteria, 62.7% of the patients had an adequate treatment response, with significant decline in BASDAI of 2.09 (*P* < 0.0001), spinal VAS of 2.92 (*P* < 0.0001) and CRP (*Z *=* *−3.048, *P* =* *0.002) at week* *16.

**Table 1.** Patient characteristics

Charactersitic	Frequency (*n* = 105)	Percentage (%)
Sex		
Male	74	70.5
Female	31	29.5
TNFi exposure		
TNFi naive	35	33.3
1 Previous TNFi	51	48.6
2 Previous TNFi	18	17.1
3 Previous TNFi	1	1

TNFi: tumour necrosis factor inhibitor.

Notably, subgroup analyses demonstrated that a substantially higher proportion of TNFi-naive patients (93.5%) exhibited improvement in BASDAI than previous TNFi therapy failures (76.8%, *P* = 0.04), creating a case to use secukinumab upfront. Spinal VAS score (85.2 *vs* 77.8%) and CRP (59.3 *vs* 46.2%) improved at 16 weeks, albeit not statistically significant.

Treatment discontinuation was seldom seen [at week* *16, 4 of 105 (3.8%)]. Two patients had colitis, one had uveitis, and in one patient secukinumab was stopped owing to inefficacy. Overall, 12.4% (*n* = 13) of patients reported an adverse event, with the most common being upper respiratory tract infection (2.9%, *n* = 3). Other reported events were lichen planus, facial rash, paraesthesia, oral candidiasis and fungal infection (Supplementary Table S1, available at *Rheumatology Advances in Practice* online).

In conclusion, the rate of treatment response in this study is comparable to MEASURE 1 and 2 ASAS 20 response [3] (although not like for like) after 16 weeks. The average change in mean BASDAI among these real-life patients, after 16 weeks, is also similar to what was obtained during a trial [3]. Report of adverse events in our audit was, however, much lower (MEASURE 1 = 68%, MEASURE 2 = 61%) [3]. Although this might be a true reflection of secukinumab adverse events in real life, it is also possible that, this being a retrospective record-based review, minor transient events were not duly captured/documented during patients’ follow-up appointments.

The numbers of patients studied are not large enough for an objective logistic regression to eliminate confounders. A longer follow-up study is planned as part of a follow-up to this study.

This real-life evaluation of secukinumab across multiple centres in the UK has demonstrated a clear benefit in its efficacy and safety in AS treatment, especially in TNFi-naive patients.

## Supplementary Material

rkad029_Supplementary_Data

## Data Availability

The data underlying this article is available and will be shared on reasonable request to the corresponding author.
