# Supplementary material for: Effectiveness and safety of secukinumab in ankylosing spondylitis: real-life data from Midlands Ankylosing Spondylitis Collaboration (MASC)
Source: Rheumatol Adv Pract. 2023 Mar 8;7(1):rkad029. doi: 10.1093/rap/rkad029 (PMC10023240; doi:10.1093/rap/rkad029)
Supplement: rkad029_Supplementary_Data [file rkad029_supplementary_data.docx]

**Supplementary Data S1. Detailed methodology**

Descriptive statistical analyses and inter-group comparisons were conducted using the commercially available statistical package for social sciences (SPSS) IBM version 22.0 analytical software.

A Q-Q plot demonstrated that all data was parametric in nature, apart from CRP which was subsequently log-transformed to ensure normal distribution for bivariate and sub-group analysis (CRP average is reported as median and interquartile range). Statistical significance was set at <0.05. A paired t-test was applied to calculate two-tailed significance of absolute mean difference in BASDAI and spinal VAS at baseline and week-16. Wilcoxon Signed Ranks Test was used to assess change in CRP.

Patients with missing mandatory variables, including baseline or week-16 BASDAI, CRP or spinal VAS scores were excluded from the bivariate statistical analyses. Sub-group analyses were then conducted to identify differences between patients who had previously been exposed to TNFi for AS and those who were TNFi naive.

For univariate analysis, all patients were included.

Only 81, 87 and 89 patients had documented Pain VAS, BASDAI and CRP (respectively) at baseline and week 16. Hence, only these numbers were included in the bivariate and sub-group analyses.

**Supplementary Table S1. Adverse events frequency**

| **Adverse events** | **Frequency**  **n (%)** |
| --- | --- |
| None | 90 (85.7) |
| Missing | 2 (1.9) |
| Upper respiratory tract infection | 3 (2.9) |
| Colitis | 2 (1.9) |
| Fungal infection | 2 (1.9) |
| Facial rash | 1(0.9) |
| Lichen planus | 1 (0.9) |
| Oral candidiasis | 1 (0.9) |
| Paraesthesia | 1 (0.9) |
| Tuberculosis | 1 (0.9) |
| Uveitis | 1 (0.9) |
| Lower respiratory tract infection | 1 (0.9) |
| Mood changes | 1 (0.9) |

***2 patients documented more than one adverse event.***
